# Intergenerational Transmission of Corporal Punishment: A Scoping Review

**DOI:** 10.1093/geroni/igaf122.3300

**Published:** 2025-12-31

**Authors:** Ying Xu, Xiafei Wang, Merril Silverstein

**Affiliations:** Syracuse University, Syracuse, New York, United States; Syracuse University, Syracuse, New York, United States; Syracuse University, Syracuse, New York, United States

## Abstract

Numerous studies have investigated the impacts of corporal punishment (CP) within families. However, there has been limited research on how these practices are transmitted across generations. This scoping review synthesized 18 peer-reviewed articles published between 2000 and 2023 from four databases, including PsycINFO, PubMed, PsycNet, and ProQuest. The findings revealed that 16 out of 18 studies showed a significant positive correlation in the intergenerational transmission of CP. Among them, nine studies indicated the intergenerational transmission of perpetrating CP, while seven found individuals with childhood CP experience would approve CP. Conversely, only one study suggested a negative association, indicating adults experienced childhood CP were less likely to use it on their own children. Additionally, one study found no significant impact of harsh mothering on later harsh parenting behaviors. These mixed findings highlight the intricate relationship between childhood CP experiences and adult disciplinary attitudes and behaviors. Future studies should prioritize longitudinal research and culturally sensitive approaches to better understand the mechanisms underlying CP transmission and develop effective strategies to break this cycle across generations.

